# Cysteine Is the Only Universally Affected and Disfavored Proteomic Amino Acid under Oxidative Conditions in Animals

**DOI:** 10.3390/antiox13030267

**Published:** 2024-02-22

**Authors:** Mario Schindeldecker, Bernd Moosmann

**Affiliations:** 1 Evolutionary Biochemistry and Redox Medicine, Institute for Pathobiochemistry, University Medical Center of the Johannes Gutenberg University, 55128 Mainz, Germany; mario.schindeldecker@unimedizin-mainz.de; 2 Institute for Pathology, University Medical Center of the Johannes Gutenberg University, 55131 Mainz, Germany; 3 Institute for Quantitative and Computational Biosciences, Johannes Gutenberg University, 55128 Mainz, Germany

**Keywords:** ageing, free radical, one-electron oxidation, peroxidation, protein oxidation, thiyl radical

## Abstract

Oxidative modifications of amino acid side chains in proteins are a hallmark of oxidative stress, and they are usually regarded as structural damage. However, amino acid oxidation may also have a protective effect and may serve regulatory or structural purposes. Here, we have attempted to characterize the global redox role of the 20 proteinogenic amino acids in animals by analyzing their usage frequency in 5 plausible evolutionary paradigms of increased oxidative burden: (i) peroxisomal proteins versus all proteins, (ii) mitochondrial proteins versus all proteins, (iii) mitochondrially encoded respiratory chain proteins versus all mitochondrial proteins, (iv) proteins from long-lived animals versus those from short-lived animals, and (v) proteins from aerobic, free-living animals versus those from facultatively anaerobic animals. We have found that avoidance of cysteine in the oxidative condition was the most pronounced and significant variation in the majority of comparisons. Beyond this preeminent pattern, only local signals were observed, primarily increases in methionine and glutamine as well as decreases in serine and proline. Hence, certain types of cysteine oxidation appear to enforce its proteome-wide evolutionary avoidance despite its essential role in disulfide bond formation and metal ligation. The susceptibility to oxidation of all other amino acids appears to be generally unproblematic, and sometimes advantageous.

## 1. Introduction

Chemical modifications of biological macromolecules are important challenges to cellular and organismal fitness that are involved in numerous diseases and in the biological aging process [1,2,3]. In general, biological macromolecular persistence may be achieved by different strategies, among them reduced numbers or levels of offenders to structural integrity (such as oxidants), intrinsically enhanced stability (due to altered usage of structural building blocks), or improved mechanisms of replacement and repair (such as reducing enzymes, chaperones, etc.). Regarding the structural stability of proteins to chemical oxidation [4], two opposing effects need to be separated: amino acid oxidation with overall adverse consequences, and amino acid oxidation with overall beneficial consequences. The former may arise when a major or minor route of oxidation leads to irreparable damage of the protein or its surroundings, such as during protein cysteine thiyl radical formation [5,6], which appears to account for the avoidance of mitochondrial membrane cysteines in long-lived animal species [7,8,9]. Cysteine thiyl radicals can relay damage into the interior of proteins [10] and act as chain-transfer catalysts in vivo [5]; they are incompletely scavenged by aqueous antioxidants like ascorbate and glutathione [5,6,11], and no efficient system seems to exist for their scavenging in hydrophobic environments [6,12]. Thus, cysteine one-electron oxidation or a related type of oxidation may account at least in part for the very low degree of conservation of solitary surface cysteine residues, as opposed to paired cysteine residues, in the general proteome [13,14], since disulfides are much more resistant to one-electron oxidation and less damaging in the one-electron oxidized state than free thiol groups [6,11,15].

In contrast, other forms of amino acid oxidation may have systemically beneficial consequences, especially when the individually effectuated chemical modification is structurally rather harmless and, preferentially, readily repairable. Under these conditions, the oxidative event may have prevented potentially much more dangerous chemical damage elsewhere in the cell. For example, protein methionine oxidation to methionine sulfoxide can be repaired enzymatically [16,17] and induces only moderate structural deterioration [16,18,19], but effectively scavenges reactive species that might have triggered a more deleterious reaction cascade elsewhere. For example, respiratory chain complexes have been shown to profoundly accumulate this labile amino acid in parallel with metabolic rate and reactive oxygen species (ROS) production, clearly indicative of an adaptive antioxidant mechanism [18,20,21,22].

In the present study, we have attempted to clarify in a systematic fashion whether the two well-established examples above, mitochondrial cysteine decumulation and mitochondrial methionine accumulation, indeed constituted the major proteomic amino acid adaptations towards oxidative stress in animals, or whether they just represented showcase examples of a much wider and diverse body of potentially adaptive, quantitative changes. Hence, a testing battery of five evolutionary paradigms of persistently increased oxidative burden in animals was devised, and the corresponding proteomes were comparatively evaluated for differences in amino acid usages. The five paradigms of comparison were designated “Peroxisome” (I), “Mitochondrion” (II), “Respiratory Chain” (III), “Longevity” (IV), and “Aerobicity” (V), and they relate to oxidative stress as follows: (i) Peroxisomes have long been recognized for their high levels of hydrogen peroxide production, which locally and sometimes globally involves substantial oxidative stress despite high local concentrations of the antioxidant enzyme, catalase [23,24]. (ii) Mitochondria usually dominate cellular oxidant production due to their potentially high superoxide release from flavoenzymes and ubiquinone oxidoreductases [25,26,27]. (iii) The prime source of superoxide within mitochondria are the respiratory chain complexes of the inner mitochondrial membrane [25,26], which are by themselves in need of special antioxidant protection [18,28]. (iv) Longevity requires particular antioxidant long-term strategies of prevention and repair [1,29,30], several of which are realized through structural amino acid usage changes [7,9,12,20,31,32,33]. (v) As opposed to anaerobic fermentation, aerobic respiration involves cellular oxygen toxicity, which is mediated by the production of reactive oxygen species (ROS) [34,35]. Through their regulatory shut-down of respiration, at least during adulthood, facultatively anaerobic parasitic helminths largely avoid oxidative stress despite the continued histological presence of mitochondria [7,36,37]. Finally, to put the obtained animal data into a wider perspective, a collection of aerobic versus anaerobic prokaryotes with well-defined oxygen requirements was sampled and analyzed accordingly.

## 2. Materials and Methods

### 2.1. Inclusion Criteria for the Analyzed Species and Proteins

A basis set for 20 animal species with well-assigned proteomes was defined from an earlier working set of 28 species [7] that had been assembled based on the following criteria: (i) an annotated nuclear-encoded proteome of >10^5^ amino acids in the NCBI RefSeq database (ncbi.nlm.nih.gov/refseq), (ii) availability of reliable lifespan data from an authoritative resource book [38] or from the peer-reviewed zoological literature, and (iii) consideration of only the most comprehensively annotated species per genus to avoid the overrepresentation of single genera. For the current study, the additional criterion of >10^2^ annotated mitochondrially imported protein sequences in NCBI RefSeq was adopted. Partial sequences and isoforms were generally excluded during proteome assembly; however, no other restrictions were applied. Peroxisomal proteins for each species were obtained from the curated Uniprot database (uniprot.org). The resulting species list and the evaluated numbers of proteins are provided in Table 1.

An extended animal species set of 218 species (chordates and arthropods) with sequenced and annotated mitochondrial genomes was compiled as described [7]. Lifespan data of these species were predominantly taken from the same resource as before [38], with the exception of the amphibians, for which another reference work was consulted amino acid usage alterations in five p [39]. Less than 10% of the lifespans were retrieved from varying sources (scholarly articles and textbooks) as detailed in [7].

A list of 86 helminths (predominantly nematodes, platyhelminths and annelids) with sequenced and annotated mitochondrial genomes was assembled as described [8]. These invertebrate species were classified according to their free-living versus parasitic lifestyle as adults; permanent adult parasitism was adopted as the criterion for anaerobic fermentative energy generation, whereas animals with a free-living lifestyle, species feeding on plants, and ectoparasitic species reversibly feeding on alternating animal hosts were classified as aerobic [8,36,37].

Prokaryotic proteomes of the groups archaea, Gram-positive bacteria and Gram-negative bacteria were assembled according to criteria already adopted before [7], especially the characterization of the prokaryotes’ growth conditions (as aerobic or anaerobic) in the authoritative database of the “Deutsche Sammlung von Mikroorganismen und Zellkulturen” (dsmz.de/collection/catalogue/microorganisms/catalogue). Strictly aerobic, microaerophilic, and facultatively anaerobic prokaryotes were collectively classified as ‘aerobic’ because of their fundamental aerotolerance. Only one species or strain per genus was included for analysis to avoid consideration bias. For the current study, sequences were obtained from the NCBI RefSeq database. As regards the archaea, the original basis set was expanded by all further species listed in a reference resource [40], resulting in 16 anaerobes and 11 aerobes. Regarding the Gram-positive bacteria, the original basis set was expanded by additional species listed in a clinical microbiology textbook [41], yielding 18 anaerobes and 16 aerobes. Regarding the Gram-negative bacteria, the original basis set was carried over, which rendered 15 anaerobes and 33 aerobes after consideration of 2 recent species reclassifications in the NCBI RefSeq database.

### 2.2. Data Analysis

Amino acid frequency data were either analyzed by comparison of group averages, yielding effect size ratios (with ratio = 1 indicating equity and, thus, unaltered usage of an amino acid under two conditions), or by linear correlation (for various analyses of the effects of longevity). Linear correlation analysis was applied to amino acid usage ratios as determined above, calculating log-linear correlation coefficients of all 20 individual amino acid usage ratios from paradigms I, II, and III with log lifespan. Moreover, linear correlation analysis was also applied to direct amino acid frequencies, calculating log-linear correlation coefficients of all 20 individual amino acids in the respiratory chain complexes of 218 animal species with log lifespan. For the unbiased comparative ranking of lifespan associations, comparisons by group were performed in addition to these correlational analyses. To this end, the group of 20 reference animals was arbitrarily divided in two groups of 10 longer-lived (15–100 years) and 10 shorter-lived (0.05–6 years) animals at the median longevity. Amino acid percentages were then directly compared without log-transformation, as in all other paradigms.

All database sequences were numerically evaluated using customized Perl scripts (http://www.perl.org) generated with the BioPerl package (http://www.bioperl.org) for computational biology [42]. Multilinear modeling for the prediction of lifespan (as variable) by the frequencies of all 20 amino acids was performed with IBM SPSS software (version 23), adopting the concomitant inclusion of all 20 amino acids as potential predictors. The resulting fractional amino acid contributions to the final model (q values in %) are provided as an outcome.

### 2.3. Transmembrane Domain Content Correction

Normalization for identical transmembrane domain (TMD) content was applied to the comparison of peroxisomal proteins versus all proteins (paradigm I), mitochondrial proteins versus all proteins (paradigm II), and mitochondrially encoded respiratory chain proteins versus all mitochondrial proteins (paradigm III). In these analyses, different proteomes with varying TMD contents (ranging from 2% to 53% in *Homo sapiens*, for example) were compared numerically, potentially introducing bias, especially in regard to the hydrophobic amino acids (which are generally accumulated in TMDs) and the highly polar and charged amino acids (which are generally decumulated in TMDs). To avoid any such bias, the pooled TMDs and the pooled non-TMDs from each proteome were analyzed for their amino acid usage separately and subsequently compared to the pooled TMDs and the pooled non-TMDs from the according reference proteome in the denominator. TMDs were determined with a hidden Markov model as implemented in the software application TMHMM 2.0 [43], which has been validated for the analysis of complete proteomes [44,45]. The output of TMHMM 2.0 was further processed using Perl scripts [7].

### 2.4. Body Mass and Phylogenetic Corrections

Body mass corrections were done by standard partial linear correlation. Body mass data for vertebrates were predominantly obtained from the AnAge database (genomics.senescence.info/species) [46]. Invertebrate body masses were assembled individually from zoological articles and monographs, or estimated from allometric body length-body mass relationships, as detailed in [7]. Phylogenetic interdependence corrections were realized through the determination of phylogenetically independent contrasts, followed by log-linear correlation of the obtained independent contrasts with log lifespan. Details on the construction of the phylogenetic trees can be found elsewhere [7].

### 2.5. Statistical Analysis

Quantitative comparisons were evaluated by non-parametric ANOVAs (Kruskal–Wallis tests) because the equal variance criterion for parametric ANOVAs was not met in many of the performed comparisons. ANOVAs were generally conducted with Systat SigmaPlot (version 11). For the linear correlations, Pearson correlation coefficients and *p* values (from two-sided tests) were determined with SPSS. Regarding the presentation of the data, the results of all 20 proteinogenic amino acids were listed, of which the 4 strongest effects, i.e., the 4 largest deviations from unity irrespective of the direction (up or down), were visually highlighted with color. The according *p* values of these four amino acids were also highlighted, but only if they were lower than *p* = 0.001. This low threshold was arbitrarily chosen to account for the multiple testing of 20 amino acids in all comparisons. Through this procedure, both effect size and effect significance were intended to be considered, but with a hierarchy to emphasize mean effects over distributional effects in the presentation of the results.

## 3. Results and Discussion

### 3.1. Amino Acid Usage Differences between Cellular Compartments

Within the eukaryotic cell, different compartments with substantially increased production of reactive oxygen species (ROS) can be distinguished. Specifically, peroxisomes are characterized by their notorious generation of hydrogen peroxide [24], whereas mitochondria constitute the predominant source of superoxide in the cell, which is mostly converted to hydrogen peroxide subsequently [26]. Within the mitochondria, the inner membrane-bound respiratory chain complexes are the prevailing local source of superoxide [27,47], providing a rationale for the separate consideration of the mitochondrial proteome as a whole, and of mitochondrially encoded respiratory chain complex subunits. Other sites of functional oxidant production do exist in eukaryotic cells, such as the endoplasmic reticulum with its protein folding-associated flavoprotein Ero1 [48], or the different sites of expression of the various NAPDH oxidases [49], but those were not investigated in the current study.

The aforesaid proteomes were collected for a sample of 20 reference animal species with high-quality proteomic coverage, which in addition were intended to represent a wide range of lifespans and several different phylogenetic groups, even if a predominance of mammals was unavoidable. The final species selection and the sequence numbers associated with each species and compartment are provided in Table 1.

Amino acid frequencies in all protein sets of Table 1 were determined, related to each other within each animal species, and then averaged over all species to calculate generalized usage ratios for each amino acid. The following usage ratios were inquired: peroxisomal proteins versus all proteins (paradigm I), mitochondrial proteins versus all proteins (paradigm II), and mitochondrially encoded respiratory chain proteins versus all mitochondrial proteins (paradigm III). These amino acid usage ratios are listed in Table 2, together with a statistical assessment by non-parametric ANOVA on ranks. In all tables in this work, the four most pronounced deviations from unity (i.e., ratio = 1.0 or correlation coefficient r = 0.0) are color-highlighted, together with the associated *p* value in case of significance at the *p* = 0.001 level. These thresholds were arbitrarily chosen, based on the number of comparisons (n = 20 amino acids) and the observed distribution of the data, which was non-normal in a substantial fraction of cases. For each species and paradigm, both protein sets to be compared were adjusted to the same transmembrane domain (TMD) content because the analyzed proteins sets were in some cases characterized by substantially different shares of TMDs. For example, all mitochondrial proteins had about 5% TMDs, whereas mitochondrially encoded proteins had about 55% TMDs, with an accordingly higher content of hydrophobic residues such as leucine and valine. In order to avoid any bias resulting from this disparity, especially any erroneous “accumulation” of hydrophobic amino acids in proteomes that were concomitantly oxidant-exposed and membrane-rich, all proteomes were individually normalized to identical TMD contents.

As evident from Table 2, the amino acid cysteine was among the four most altered amino acids in all three paradigms, and its modulation was always negative and always reached statistical significance. Concordant effects in at least two paradigms were an increase in methionine in paradigms II and III, and a loss of proline and serine in paradigms I and II. Notable effects at the level of a single paradigm were an increase of isoleucine in paradigm I, which was the strongest signal in peroxisomes, and an accumulation of asparagine as well as a loss of arginine in paradigm III (respiratory chain complexes), the latter of which occurred despite correction for equal TMD content.

Since paradigm III investigates proteomes derived from mitochondrial translation systems occasionally using a different genetic code, the associated decoding changes are also provided in Table 2. Qualitatively, methionine accumulation was related to a genetic code change that has been shown to be adaptive and causative to the accumulation [18]. In addition, arginine loss and tryptophan gain were also associated with the deviant genetic decoding in animal mitochondria, which use two codons (UGA and UGG) throughout to encode tryptophan instead of one codon (the canonic UGG codon). Moreover, 13 out of the 20 selected animal species do not encode arginine by AGA and AGG, reducing the number of arginine codons from 6 canonic codons to 4 codons. Both changes were apparently not buffered by compensatory changes in codon usage, as they both contributed to significantly altered amino acid frequencies at the protein level (Table 2). After a long debate on the origin of the modern genetic code variants [50,51], there is compelling evidence now to indicate that deviant genetic codes like those in animal mitochondria primarily evolve to match the amino acid requirements of the encoded proteins [18,51,52] rather than having their foundation at the DNA or RNA level. Interestingly, the loss of the AUA codon for Ile (in 19 out of 20 species) was evidently compensated, and did not result in an overall loss of isoleucine at the protein level. Without compensation, one would have expected such an isoleucine loss if methionine accumulation were the selective driver of the AUA Ile-to-Met transition. No attempts were made in this work to formally adjust for coding differences across species because the final outcome at the protein level was regarded as the relevant biochemical parameter to consider when studying effects of redox reactivity at the protein level. The individual mechanisms by which amino acid changes are installed over evolutionary time in vivo are certainly interesting and multi-dimensional (e.g., individual mutations, collective GC-content shifts, genetic code changes), but without immediate relevance to the interpretation of the final number of reactive chemical groups in proteins, which was the primary aim of the current comparative investigation.

**Figure 1:**
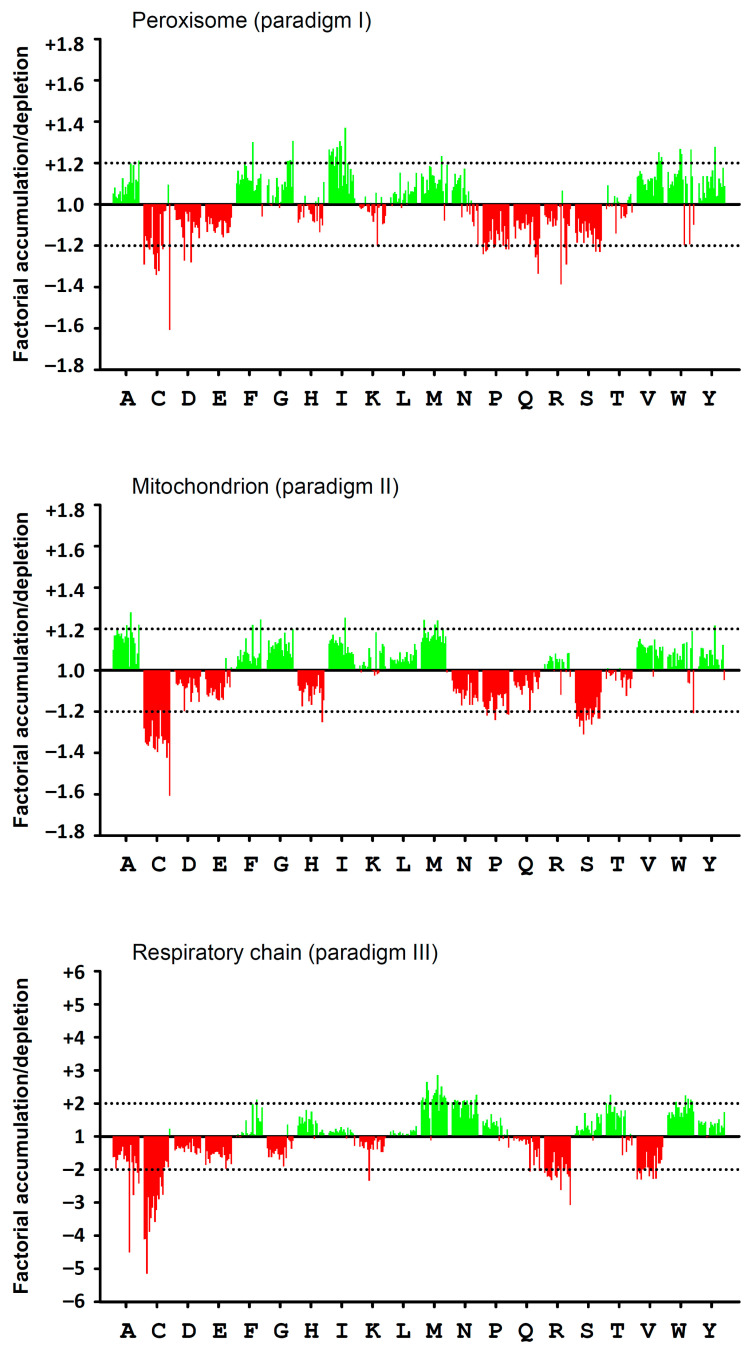
Graphical representation of the factorial accumulation (positive values, green color) or depletion (negative values, red color) of each amino acid in paradigms I, II and III. Each of the 20 investigated animal species in these paradigms is represented by a single line within every 20-line stack per amino acid. Animals were sorted by decreasing longevity from left to right, as per Table 1 (i.e., from *Homo sapiens*, left, to *Caenorhabditis elegans*, right).

A graphical representation of the quantitative data of Table 2 is provided in Figure 1. Therein, fractional increases or decreases are plotted for each amino acid as a bar stack, in which each bar (as red or green line) represents 1 (out of 20) animal species. The 20 animal species were sorted by decreasing lifespan from left to right analogously, as listed in Table 1. Several aspects in this comparison across paradigms and amino acids are noteworthy. First, amino acid usage changes in mitochondrially encoded proteins (versus all mitochondrial proteins, paradigm III) were generally more pronounced, often reaching a factorial accumulation/depletion by a factor of 2 (or ½). In the other paradigms, the approximate equivalence was a factorial accumulation/depletion by a factor of 1.2 (or 1/1.2). Still, interspecies variation (i.e., the variability within each bar stack) was largely unrelated to the degree of accumulation/depletion, but it appeared to be somewhat higher in peroxisomes, which is likely attributable to the smaller number of proteins in several representatives of this groups (Table 1). The most interesting outcome of the vertical comparison of the three plotted paradigms is yet their similarity, particularly as regards the prototypically redox-active amino acids cysteine, methionine, tyrosine and tryptophan: cysteine was strongly decreased (red color in Figure 1), whereas methionine, tyrosine and tryptophan were increased throughout (green color in Figure 1). As further discussed below, this behavior matches well with the inferred functional consequences of amino acid (one-electron) oxidation in (membrane) proteins as derived from chemical reaction rate constants [12].

### 3.2. Amino Acid Usage Alterations in Relation to Longevity

In the majority of cases shown in Figure 1, amino acid changes were similar across species and, thus, lifespans; in other words, most bar stacks had a more or less horizontal upper or lower borderline. However, the amino acid cysteine in paradigm III displayed a borderline that pronouncedly inclined to the left, denoting a more severe usage bias in long-lived animal species. This signal has been studied before [7]; however, a formal comparison with all other amino acids and across paradigms has been lacking. Hence, as a fourth paradigm, the correlation of each of the amino acid accumulation/depletion signals with lifespan was investigated.

Table 3 presents the results obtained by a linear correlation analysis of double log-transformed data (log amino acid usage ratio versus log lifespan in years). In the three paradigms I–III, several amino acids reached correlation coefficients of 0.5 or higher in absolute values. However, only one signal in paradigms I and II reached statistical significance, namely asparagine, which was increased in peroxisomal proteins of long-lived species, as directly visible from Figure 1. In all other cases, apparently inclined bar stacks turned out to be insignificantly associated with longevity due to inter-species variation or small effect sizes. In mitochondrially encoded proteins, four amino acid alterations were significantly associated with longevity, namely cysteine loss, which was the strongest correlation, followed by threonine, glutamine and proline (Table 3), all of which were increased with longevity. Of note, selection for threonine has been described before as a longevity-related adaptation in long-lived mammals and explained with structural, non-redox properties of this amino acid in transmembrane domains [32,45]. The same studies have also noted the negative cysteine signal.

Parametric longevity correlations offer the potentially insightful opportunity to compare molecular correlations of interest to other inherent correlatives of longevity such as body mass, which is the undisputed master predictor of lifespan in animals [53], or phylogenetic co-segregation. Both correlatives can be corrected for, either to calculate the degree by which a molecular correlation is better than the master correlation with body mass, or to calculate the degree by which a molecular trait is better correlated with longevity than expected from species phylogeny. These corrections are usually realized by partial correlation, or by phylogenetically independent contrast analysis [54] followed by correlation [7,20,45]. Still, it is important to note that even if a correlation were not significant any more after any of these corrections, it may still be causally and relevantly involved in the limitation of longevity, e.g., in the standard case of molecular co-segregation [55].

To adopt both of these corrections, body masses were collected, and a phylogenetic tree was assembled for the animal species under investigation, as described in [7]. Both corrections were applied to the paradigm with the strongest signals at baseline, namely longevity within the respiratory chain complex signals (denoted paradigm IVc in Table 3). The resulting corrected correlation coefficients and the ensuing *p* values are provided in Table 4. As a further analysis presented in Table 4, a multilinear predictive modelling of lifespan using all 20 amino acids was performed, followed by inspection of the relative contribution of each amino acid to the generated model (as q value in %). Finally, to better consider effect sizes (which are sometimes obfuscated in correlation analyses), simple binomial ratios were calculated after division of the 20 animal species into 2 groups of 10 species each, encompassing short-lived (0.05–6 years) and long-lived (15–100 years) animals (Table 1).

Body mass correction led to substantially reduced correlation coefficients throughout, turning all single amino acid associations statistically insignificant (Table 4). Phylogenetic correction had a comparable effect, with the exception of cysteine, the negative association of which with longevity was preserved. Notably, cysteine’s correlation coefficient (r = −0.791) still came close to the correlation coefficient of body mass with longevity (r = −0.886), similar to before in the uncorrected data. This is an intriguing result because a single amino acid in a rather small proteome would not be expected to predict a complex trait like lifespan almost equally well as the complex reference trait body mass, which is inevitably linked to lifespan on multiple levels. After all, the building of a large body requires time due to the inherent temporal limits of cell division and bioenergetic uptake. Cysteine also emerged as prime lifespan-associated amino acid from multilinear modelling, contributing 43% to the final correlation, followed by asparagine (12%). In the effect size calculations, cysteine again ranked as the most differentially used amino acid in long-lived versus short-lived species, followed by threonine (increased), glutamine (increased), and phenylalanine (decreased). Of the latter amino acids, glutamine and threonine had also been marked as significantly altered in the same direction in the correlational analysis (Table 4).

The above observations were put on probation by examining a second larger dataset of 218 animal species with known body mass and phylogenetic positioning. Due to the lack of fully sequenced proteome data for the majority of these species, only the smaller mitochondrially encoded proteomes were assessed (i.e., the numerators of the fractions represented by paradigm IVc). The results in Table 5 demonstrate a large overlap with the findings obtained before in the internally controlled 20-species reference set. Specifically, the negative correlation of cysteine with lifespan was reproduced, as were the positive correlations of proline and threonine. In addition, a negative phenylalanine association emerged that had just failed to reach significance in the 20-species cohort. Uniquely, the cysteine-lifespan correlation remained significant after corrections for body mass and lifespan as reported in [7], and hence in all three correlational analyses (Table 5). Proline was the second-best predictor of lifespan in this dataset, as it reached significance in two out of three correlations and ranked second in the multilinear model. Cytosolic proline in yeast [56], as well as several other soluble amino acids in animals, have been shown to be related to longevity or cellular senescence before [57,58]. In plants, cytosolic proline may act as an indirect quencher of different oxidants [59]. Still, it is unclear in how far such data for soluble molecules may be extrapolated to the much less dynamic amino acid contents of proteins. In fact, as exemplified for cysteine, soluble chemical groups may exhibit exactly converse associations with longevity than the same chemical groups in macromolecules because the sacrifice of the former may help protect the latter [6,12].

### 3.3. Amino Acid Usage Adaptations Linked to an Aerobic Lifestyle

Parasitic helminths have long been known to avoid oxygen-dependent ATP generation and largely depend on anaerobic fermentation after infection of their host, even in cases in which oxygen partial pressures would be more than sufficient for aerobic respiration [36,37]. The most plausible origin of this initially unexpected behavior is the potential toxicity arising from reactive oxygen species (ROS) produced during respiration, to which many parasitic species are poorly shielded when compared to their vertebrate hosts [60,61]. Notably, infections with facultatively anaerobic eukaryotes are in many instances effectively treated with prooxidative drugs such as artemisinin [62], metronidazole [63] or chloroquine [64].

To examine potential differences in the proteomic composition of aerobic and facultatively anaerobic animal species, a collection of mitochondrially encoded proteomes was assembled, encompassing 55 facultatively anaerobic (parasitic) and 31 aerobic (free-living) species predominantly of the nematode and platyhelminth phyla [8]. Table 6 shows the outcome of this comparison. Global cysteine avoidance in aerobes was the most distinct difference in terms of effect size, whereas valine avoidance and glutamine preference were the most significant signals in statistical terms. Beyond these three amino acids, several other amino acids were characterized by usage differences in aerobic versus anaerobic species of more than 50% in the analyzed highly homologous proteomes (i.e., mitochondrial respiratory chain complexes). This unexpected finding prompted us to expand the comparative investigation of the proteomic footprints of aerobicity to larger proteomes. With only four fully sequenced proteomes of aerobic helminths as per the end of 2023, and two of them being of the same genus, *Caenorhabditis*, total proteomic comparison of helminths was regarded unreliable. Hence, three groups of prokaryotes with well-defined oxygen preferences were collected to augment the data generated in helminths. More specifically, groups of approximately 30–50 archaea (paradigm VI), Gram-positive bacteria (paradigm VII) and Gram-negative bacteria (paradigm VIII) were classified according to their oxygen preferences, followed by analysis of their total proteomic amino acid usage patterns. The score provided in Table 7 indicates that effect sizes and significance levels were smaller than in the helminths, even if certain signals were in fact recapitulated. Cysteine avoidance in Gram-positive bacteria pursuing aerobic respiration was the most pronounced and significant change seen across all groups, followed by glutamine accumulation in Gram-negative bacteria. Aerobic bacteria also elicited increased tryptophan usage. As an entirely new signal absent in animals, lysine avoidance was noted in all groups of aerobic prokaryotes. Still, neither tryptophan nor lysine reached statistical significance in any individual group.

Despite the high diversity of prokaryotes in terms of their metabolic activities, various observations support their usefulness as evolutionary indicators of oxidative stress. For instance, strains of *Escherichia coli* have been shown to respond to oxidative stress through adaptations in their structural proteomes [65]. Significant differences have also been noted for the proteomic amino acid usage of 295 aerobic versus anaerobic prokaryotes after correction for phylogenetic interdependence, namely increases in tryptophan and threonine and a decrease in cysteine [66]. Comparisons of *Escherichia coli* with (highly radioresistant) *Deinococcus radiodurans* have suggested that lower cysteine and methionine contents were decisive for the resistance of the latter species, and that lysine increased the accessibility of neighboring redox-reactive residues to oxidants, leading to its elimination. Tryptophan and tyrosine were likewise significantly enriched in *Deinococcus radiodurans* [67]. Overall, these patterns clearly resemble the trends listed in Table 7.

### 3.4. Relation of the Observed Amino Acid Usage Differences to Amino Acid Redox Reactivity

To enable a comparative survey of the detected amino acid usage alterations in the individual paradigms, the measured effect sizes of all changes were ranked from 1–20, with 1 being the most heavily altered amino acid in each comparison and 20 being the least altered amino acid. The resulting score for the animal paradigms I–V is provided in Table 8, with increases in green and decreases in red; the score for the prokaryotes is provided in Table 9. Amino acids that were regarded as potentially altered in two or more paradigms (and thus highlighted in Table 1, Table 2, Table 3, Table 4, Table 5 and Table 6) are further discussed in the following.

**Cysteine** avoidance in mitochondrial proteins has been described in different datasets before [7,8], but its extent, generalizability and relative relevance compared to other amino acid changes under oxidative stress on evolutionary timescales have not been systematically evaluated. From the present meta-examination, it becomes clear that the general susceptibility of protein cysteine to oxidation is in fact the primary subject of proteomic adaptations to persistently increased levels of reactive oxygen species (ROS). Table 10 provides an overview of the effectuated quantitative amino acid frequency changes for cysteine and the prototypically redox-active amino acids methionine, tryptophan and tyrosine. In three unrelated paradigms (III, V and VII), cysteine was decreased by approximately 50% on a proteome-wide scale, representing the largest effect size of all amino acids.

Independently of specific redox assumptions, a very low degree of conservation of isolated, solitary surface cysteines has been evidenced before, which was contrasted by maximum conservation of potentially disulfide-forming cysteine pairs [13,14]. Even if no separate analysis of solitary versus paired cysteines was attempted in the current investigation, it is highly plausible to suppose that the main component of the redox-related cysteine bias described herein is attributable to solitary cysteines. If this were correct, the global cysteine losses of Table 10 would imply even higher fractional losses when regarding only solitary cysteines. The large frequency changes obtained in paradigms III and IV provide support for this interpretation because both paradigms relate to respiratory chain complexes that do not possess any disulfide bridges and harbor only one conserved cysteine pair that ligates copper in respiratory chain complex IV [7].

Since redox-related cysteine avoidance, as described herein, likely affects solitary surface cysteines [13] and generally unpaired transmembrane cysteines [8] in a selective fashion, it has been proposed that the one-electron oxidation of cysteine thiol groups to thiyl radicals may mediate these features of proteomic adaptation [6]. Detailed analyses of the reaction rate constants of thiyl radicals in the context of lipid peroxidation have supported this view exemplarily [12]. In the current survey, cysteine was the most or second-most heavily affected amino acid in 7 out of 8 paradigms, with Gram-negative bacteria (paradigm VIII) being the only exception (Table 7 and Table 9). As Gram-negative bacteria usually possess the capacity to synthesize ample amounts of dedicated antioxidant thiols like glutathione [68], which contrasts archaea and Gram-negative bacteria, our data strongly suggest that the accumulation of soluble thiols in Gram-negative bacteria enabled the continued high-frequency usage of cysteine as protein structural component despite rising oxygen concentrations or intensified oxygen utilization [69]. This interpretation is chemically plausible, as soluble thiols show very high reactivity towards thiyl radicals to generate mostly harmless, mixed disulfide radicals [6,11].

Adaptive increases in proteomic **methionine** in oxidant-exposed proteomes have also been observed before, with the conclusion that such increases in methionine essentially mark particularly vulnerable, biologically precious proteins rather than sites with the highest oxidant production [18,21]. This idea fits to the observed antioxidant chemistry and reactivity of methionine, which reacts preferentially with non-radical species by two-electron reduction rather than blunting radical species through one-electron reduction [12]. No association of methionine with longevity was observed in any of our datasets (Table 3, Table 4 and Table 5). This stands in partial contrast to earlier studies focusing on mammals, in which, after phylogenetic correction, an otherwise invisible signal had emerged [19,20]. Potentially, methionine-longevity adaptations occur exclusively in higher vertebrate clades. Notably, negative changes in the soluble level of methionine and various metabolites of the methionine degradation pathway have been found to be prominent biochemical predictors of longevity and stress resistance [33,58,70]. Even if soluble amino acid concentrations should not have any immediate influence on their usage in genetically encoded proteins, a coevolution of both properties is clearly conceivable [33,70].

**Serine** was significantly reduced in two paradigms and lowered by trend in several other comparisons (Table 2, 5 and 6). Even if serine is not regarded as a typical redox-active amino acid, it has been shown to be one of the fastest amino acids to react with neighboring thiyl radicals, carrying their detrimental reactivity into the interior of the protein [6,10]. Potentially, this secondary effect underlies the avoidance of serine in specific situations. In interesting contrast, the chemically related amino acid threonine was unaltered throughout, except for paradigms investigating respiratory chain complexes and their adaptations towards increased longevity (Table 4 and Table 5). Strikingly, the phenomenon of an adaptive respiratory chain threonine increase in long-lived animals has been characterized in detail before, proposing a structural rather than reactivity origin of this alteration in terms of a selective hydrophobicity modulation [31,45]. The singularity and unusual strength of the current threonine signal underline this view.

Significant avoidance of **proline** was observed in two paradigms (I and II), but proline was otherwise unchanged or increased in all other comparisons, the latter especially in relation to longevity (Table 4 and Table 5). The interpretation of these findings is unclear for now. Proline shows a relatively high degree of hydroperoxide formation after attack by hydroxyl radicals [4]. However, comparable quality targets of hydroperoxide modification were either unchanged (i.e., Leu, which was the most inert amino acid across all paradigms in this study), or modulated in opposite directions (i.e., Val, showing substantial peaks up (paradigm I and II) and down (paradigm III and V)), or selectively increased (i.e., Ile (paradigm I)). Hence, the changes in proline and the branched-chain hydrophobic amino acids may rather be structural-individual rather than stemming from general chemical reactivity patterns, and may perhaps also be attributable to selective hydrophobicity modulation [31,45]. Of note, together with methionine, valine was among the most meaningful amino acids that, in respiratory chain sites of 324 vertebrates, individually predicted longevity [71]. In any case, the high volatility of valine compared to the structurally related leucine (Table 8) is certainly interesting and requires further study. The same applies to the so-far unexplained negative correlation of the soluble amino acid levels of proline with lifespan [58].

Finally, asparagine and **glutamine** were significantly increased or showed indicative trends in a number of evaluations, including peroxisome-longevity (paradigm IVa), respiratory chain-longevity (paradigm IVc), and aerobicity (paradigm V) (Table 3, Table 4 and Table 6). By most measures, asparagine and glutamine are hardly oxidation-prone amino acids [4,22,72], such that their accumulation in peroxisomal proteins of longer-lived animals is very likely unrelated to their redox properties. Somewhat paradoxically, though, asparagine is one of the most easily isomerized and hydrolyzed amino acids, resulting in the formation of isoaspartate and D-aspartate in proteins [73]. Both isoaspartate and D-aspartate are well-established markers of protein aging and reflect organismal aging in particularly long-lived proteins such as eye lens crystallins [74]. Hence, one would have rather expected the avoidance of asparagine and perhaps other hydrolysis-prone amino acids in proteins of long-lived species. As a bona fide interpretation of this discrepancy, spontaneous isomerization of asparagine and glutamine may have an only softly adverse effect on organismal longevity that is perhaps readily overcompensated by certain structural advantages of the amino acids themselves. One of these advantages could be their chaotropic solubility-enhancing activity on protein surfaces [22], preventing oxidized or otherwise denatured proteins from aggregation, a common cause of age-related disease.

## 4. Conclusions

In a multi-panel comparative analysis of global amino acid usage in animal proteomes exposed to different levels and types of intrinsic oxidative stress, we have identified cysteine as the most universally and pronouncedly affected amino acid. In contrast to the observed avoidance of cysteine, the other canonically redox-active amino acids methionine, tyrosine and tryptophan were widely increased, but they only reached significance in a limited number of comparisons, notably methionine in mitochondrial proteomes. As regards all other amino acids, relevant changes in more than one comparison were restricted to increases in glutamine and decreases in serine and proline. For these cases, the literature data and specificity considerations mostly suggest non-redox or only indirectly redox-related causes for their altered occurrence. Cysteine is the only amino acid the presence of which in proteins appears to be evolutionarily suppressed under conditions of oxidative stress.

## Figures and Tables

**Table 1:** The investigated animals and their lifespans, body masses, and sampled sequence numbers.

Species	Phylogenetic Group	Lifespan (Years)	Body Mass (kg)	Analyzed Sequences		
				Full proteome	Mitochondrion	Peroxisome
*Homo sapiens*	Mammalia	100	70	15,072	978	238
*Equus caballus*	Mammalia	62	1000	16,148	283	100
*Pan troglodytes*	Mammalia	60	45	14,349	376	168
*Macaca mulatta*	Mammalia	35	8	15,939	314	145
*Bos taurus*	Mammalia	30	750	28,590	749	110
*Canis familiaris*	Mammalia	30	40	14,903	429	227
*Gallus gallus*	Aves	30	2.6	15,525	301	101
*Strongylocentrotus purpuratus*	Echinoidea	20	0.1	25,696	309	29
*Oryctolagus cuniculus*	Mammalia	18	1.8	14,808	269	90
*Cavia porcellus*	Mammalia	15	0.73	16,419	204	59
*Monodelphis domestica*	Mammalia	6	0.11	16,593	273	46
*Mus musculus*	Mammalia	6	0.021	21,551	1186	188
*Apis mellifera*	Insecta	5	9 × 10^−5^	8529	204	19
*Danio rerio*	Pisces	5	5 × 10^−4^	25,049	588	62
*Rattus norvegicus*	Mammalia	4	0.3	26,060	799	158
*Ciona intestinalis*	Ascidiae	2	1 × 10^−4^	11,653	203	25
*Tribolium castaneum*	Insecta	2	2.5 × 10^−6^	9340	193	55
*Drosophila melanogaster*	Insecta	0.15	1 × 10^−6^	9748	162	45
*Anopheles gambiae*	Insecta	0.07	2.5 × 10^−7^	11,200	126	36
*Caenorhabditis elegans*	Nematoda	0.05	2 × 10^−9^	23,865	219	39

**Table 2:** Numerical evaluation of the amino acid usage ratios in paradigms I, II, and III.

Amino Acid	Peroxisome (Paradigm I)		Mitochondrion (Paradigm II)		Respiratory Chain (Paradigm III)		
	Ratio	*p*	Ratio	*p*	Ratio	*p*	Decoding *
A	**1.09**	3 × 10^−4^	**1.15**	3 × 10^−5^	**0.57**	1 × 10^−7^	
C	**0.86**	3 × 10^−5^	**0.74**	1 × 10^−7^	**0.41**	1 × 10^−7^	
D	**0.91**	8 × 10^−5^	**0.93**	4 × 10^−4^	**0.74**	2 × 10^−7^	
E	**0.91**	3 × 10^−7^	**0.92**	2 × 10^−6^	**0.63**	6 × 10^−8^	
F	**1.12**	2 × 10^−6^	**1.08**	5 × 10^−5^	**1.28**	1 × 10^−2^	
G	**1.09**	9 × 10^−5^	**1.12**	4 × 10^−4^	**0.73**	1 × 10^−6^	+AGA, +AGG (1/20)
H	**0.97**	1 × 10^−2^	**0.90**	3 × 10^−6^	**1.36**	3 × 10^−5^	
I	**1.19**	1 × 10^−4^	**1.12**	2 × 10^−3^	**1.12**	4 × 10^−3^	−AUA (19/20)
K	**0.97**	7 × 10^−2^	**1.04**	2 × 10^−1^	**0.79**	4 × 10^−5^	−AAA (1/20)
L	**1.05**	1 × 10^−2^	**1.06**	6 × 10^−3^	**1.11**	2 × 10^−7^	
M	**1.11**	2 × 10^−4^	**1.16**	1 × 10^−6^	**2.14**	2 × 10^−7^	+AUA (19/20)
N	**1.03**	2 × 10^−1^	**0.90**	8 × 10^−3^	**1.93**	9 × 10^−8^	+AAA (1/20)
P	**0.86**	3 × 10^−4^	**0.86**	3 × 10^−4^	**1.28**	3 × 10^−3^	
Q	**0.88**	9 × 10^−6^	**0.93**	4 × 10^−4^	**0.81**	2 × 10^−5^	
R	**0.92**	2 × 10^−3^	**1.03**	2 × 10^−2^	**0.49**	6 × 10^−8^	−AGA, −AGG (13/20)
S	**0.87**	2 × 10^−7^	**0.82**	6 × 10^−8^	**1.29**	3 × 10^−6^	+AGA, +AGG (6/20)
T	**0.99**	6 × 10^−1^	**0.97**	1 × 10^−1^	**1.48**	7 × 10^−3^	
V	**1.14**	1 × 10^−6^	**1.10**	1 × 10^−6^	**0.53**	6 × 10^−8^	
W	**1.10**	2 × 10^−3^	**1.05**	3 × 10^−2^	**1.81**	6 × 10^−8^	+UGA (20/20)
Y	**1.10**	4 × 10^−3^	**1.06**	1 × 10^−2^	**1.33**	6 × 10^−6^	

*p* Values derive from non-parametric ANOVA on ranks analyses. Red color highlights the 4 most pronounced effect sizes among the 20 amino acids and their significance. The according *p* values were also highlighted if a predefined threshold of *p* < 0.001 was reached. Amino acids are given in single-letter code. * When amino acids were encoded in mitochondria by more or fewer codons than in the standard genetic code, those codons are given in this column. The numbers in brackets indicate how many of the examined 20 animal species use the non-standard decoding.

**Table 3:** Quantitative correlation with lifespan of the signals in paradigms I, II and III (paradigm IV).

Amino Acid	Longevity within Paradigm I (Paradigm IVa)		Longevity within Paradigm II (Paradigm IVb)		Longevity within Paradigm III (Paradigm IVc)	
	r	*p*	r	*p*	r	*p*
A	**−0.598**	5 × 10^−3^	**0.220**	4 × 10^−1^	**0.370**	1 × 10^−1^
C	**−0.166**	5 × 10^−1^	**0.523**	2 × 10^−2^	**−0.888**	2 × 10^−7^
D	**0.159**	5 × 10^−1^	**0.220**	4 × 10^−1^	**0.190**	4 × 10^−1^
E	**0.064**	8 × 10^−1^	**−0.505**	2 × 10^−2^	**0.215**	4 × 10^−1^
F	**0.365**	1 × 10^−1^	**−0.175**	5 × 10^−1^	**−0.651**	2 × 10^−3^
G	**−0.655**	2 × 10^−3^	**−0.082**	7 × 10^−1^	**−0.436**	5 × 10^−2^
H	**−0.096**	7 × 10^−1^	**0.350**	1 × 10^−1^	**0.535**	2 × 10^−2^
I	**0.593**	6 × 10^−3^	**0.616**	4 × 10^−3^	**0.502**	2 × 10^−2^
K	**0.349**	1 × 10^−1^	**−0.375**	1 × 10^−1^	**−0.131**	6 × 10^−1^
L	**−0.493**	3 × 10^−2^	**−0.636**	3 × 10^−3^	**−0.554**	1 × 10^−2^
M	**0.156**	5 × 10^−1^	**0.485**	3 × 10^−2^	**−0.163**	5 × 10^−1^
N	**0.712**	4 × 10^−4^	**0.353**	1 × 10^−1^	**0.072**	8 × 10^−1^
P	**−0.018**	9 × 10^−1^	**0.020**	9 × 10^−1^	**0.669**	1 × 10^−3^
Q	**0.671**	2 × 10^−3^	**−0.154**	5 × 10^−1^	**0.686**	8 × 10^−4^
R	**0.099**	7 × 10^−1^	**0.053**	8 × 10^−1^	**0.321**	2 × 10^−1^
S	**0.510**	2 × 10^−2^	**−0.369**	1 × 10^−1^	**−0.484**	3 × 10^−2^
T	**−0.012**	1 × 10^−0^	**0.490**	3 × 10^−2^	**0.697**	6 × 10^−4^
V	**−0.352**	1 × 10^−1^	**0.219**	4 × 10^−1^	**−0.562**	1 × 10^−2^
W	**0.236**	3 × 10^−1^	**0.241**	3 × 10^−1^	**−0.474**	3 × 10^−2^
Y	**−0.257**	3 × 10^−1^	**0.223**	3 × 10^−1^	**−0.161**	5 × 10^−1^

*p* Values derive from linear correlations of double log-transformed data. Red color highlights the 4 strongest correlations among the 20 amino acids and, if applicable, their significance.

**Table 4:** Physiological analyses on paradigm IVc: correlation of longevity with amino acid usage in respiratory chain proteins versus all mitochondrial proteins.

Amino Acid	Raw			Body Mass-Corrected		Phylogeny-Corrected		Effect Size	
	r	*p*	q [%]	r	*p*	r	*p*	Ratio	*p*
A	**0.370**	1 × 10^−1^	1	**−0.099**	7 × 10^−1^	**0.063**	8 × 10^−1^	**1.22**	2 × 10^−2^
C	**−0.888**	2 × 10^−7^	43	**−0.162**	5 × 10^−1^	**−0.791**	6 × 10^−5^	**0.53**	3 × 10^−4^
D	**0.190**	4 × 10^−1^	1	**−0.150**	5 × 10^−1^	**−0.161**	5 × 10^−1^	**1.00**	5 × 10^−1^
E	**0.215**	4 × 10^−1^	9	**−0.131**	6 × 10^−1^	**0.281**	2 × 10^−1^	**1.04**	2 × 10^−1^
F	**−0.651**	2 × 10^−3^	3	**0.234**	3 × 10^−1^	**−0.170**	5 × 10^−1^	**0.74**	2 × 10^−2^
G	**−0.436**	5 × 10^−2^	5	**0.084**	7 × 10^−1^	**−0.434**	6 × 10^−2^	**0.84**	3 × 10^−1^
H	**0.535**	2 × 10^−2^	4	**−0.286**	2 × 10^−1^	**−0.011**	1 × 10^−0^	**1.17**	3 × 10^−2^
I	**0.502**	2 × 10^−2^	4	**−0.020**	9 × 10^−1^	**0.619**	5 × 10^−3^	**1.06**	3 × 10^−1^
K	**−0.131**	6 × 10^−1^	4	**−0.221**	4 × 10^−1^	**−0.375**	1 × 10^−1^	**0.92**	5 × 10^−1^
L	**−0.554**	1 × 10^−2^	0	**0.104**	7 × 10^−1^	**−0.519**	2 × 10^−2^	**0.97**	5 × 10^−1^
M	**−0.163**	5 × 10^−1^	3	**−0.285**	2 × 10^−1^	**−0.379**	1 × 10^−1^	**0.88**	2 × 10^−1^
N	**0.072**	8 × 10^−1^	12	**−0.520**	2 × 10^−2^	**−0.141**	6 × 10^−1^	**1.04**	6 × 10^−1^
P	**0.669**	1 × 10^−3^	1	**0.059**	8 × 10^−1^	**0.412**	8 × 10^−2^	**1.22**	3 × 10^−2^
Q	**0.686**	8 × 10^−4^	2	**−0.081**	7 × 10^−1^	**0.292**	2 × 10^−1^	**1.27**	1 × 10^−2^
R	**0.321**	2 × 10^−1^	1	**0.325**	2 × 10^−1^	**0.473**	4 × 10^−2^	**1.02**	6 × 10^−1^
S	**−0.484**	3 × 10^−2^	1	**−0.007**	1 × 10^−0^	**0.177**	5 × 10^−1^	**0.93**	4 × 10^−1^
T	**0.697**	6 × 10^−4^	0	**−0.247**	3 × 10^−1^	**0.145**	6 × 10^−1^	**1.52**	7 × 10^−3^
V	**−0.562**	1 × 10^−2^	4	**0.019**	9 × 10^−1^	**−0.577**	1 × 10^−2^	**0.88**	1 × 10^−1^
W	**−0.474**	3 × 10^−2^	2	**0.133**	6 × 10^−1^	**−0.028**	9 × 10^−1^	**0.91**	7 × 10^−2^
Y	**−0.161**	5 × 10^−1^	0	**−0.369**	1 × 10^−1^	**−0.564**	1 × 10^−2^	**0.98**	8 × 10^−1^
Body mass	**0.933**	2 × 10^−9^	−	**−**	−	**0.886**	5 × 10^−7^	−	−

*p* Values derive from linear correlations of double log-transformed data. For ratios, *p* values derive from ANOVA on ranks analyses. Red color highlights the 4 strongest correlations or the 4 largest effects among the 20 amino acids and, if applicable, their significance.

**Table 5:** Mitochondrially encoded amino acid usage and longevity in 218 species.

Amino Acid	Raw			Body Mass-Corrected		Phylogeny-Corrected	
	r	*p*	q [%]	r	*p*	r	*p*
A	**0.569**	5 × 10^−20^	0	**0.130**	6 × 10^−2^	**−0.022**	8 × 10^−1^
C	**−0.737**	2 × 10^−38^	39	**−0.322**	2 × 10^−6^	**−0.325**	1 × 10^−6^
D	**0.039**	6 × 10^−1^	1	**−0.074**	3 × 10^−1^	**0.088**	2 × 10^−1^
E	**0.516**	4× 10^−16^	5	**0.387**	4 × 10^−9^	**0.119**	8 × 10^−2^
F	**−0.740**	5 × 10^−39^	1	**−0.226**	1 × 10^−3^	**−0.133**	5 × 10^−2^
G	**−0.036**	6 × 10^−1^	1	**0.005**	9 × 10^−1^	**0.004**	1 × 10^−0^
H	**0.706**	4× 10^−34^	8	**0.250**	2 × 10^−4^	**0.201**	3 × 10^−3^
I	**−0.347**	2 × 10^−7^	0	**−0.129**	6 × 10^−2^	**0.057**	4 × 10^−1^
K	**−0.216**	1 × 10^−3^	7	**0.149**	3 × 10^−2^	**0.059**	4 × 10^−1^
L	**0.416**	2 × 10^−10^	0	**0.039**	6 × 10^−1^	**0.033**	6 × 10^−1^
M	**−0.255**	2 × 10^−4^	1	**−0.098**	2 × 10^−1^	**−0.007**	9 × 10^−1^
N	**−0.351**	2 × 10^−7^	7	**−0.309**	4 × 10^−6^	**−0.104**	1 × 10^−1^
P	**0.768**	1 × 10^−43^	17	**0.350**	2 × 10^−7^	**0.163**	2 × 10^−2^
Q	**0.610**	2 × 10^−23^	0	**0.096**	2 × 10^−1^	**0.005**	9 × 10^−1^
R	**0.475**	2 × 10^−13^	4	**0.077**	3 × 10^−1^	**−0.052**	4 × 10^−1^
S	**−0.620**	2 × 10^−24^	0	**−0.105**	1 × 10^−1^	**−0.036**	6 × 10^−1^
T	**0.747**	4 × 10^−40^	1	**0.236**	5 × 10^−4^	**0.167**	1 × 10^−2^
V	**−0.183**	7 × 10^−3^	1	**−0.022**	7 × 10^−1^	**0.024**	7 × 10^−1^
W	**0.329**	7 × 10^−7^	0	**0.111**	1 × 10^−1^	**0.172**	1 × 10^−2^
Y	**−0.518**	3 × 10^−16^	9	**−0.393**	3 × 10^−9^	**−0.064**	3 × 10^−1^
Body mass	**0.868**	2 × 10^−67^	-	-	-	**0.555**	6 × 10^−19^

*p* Values derive from linear correlations of double log-transformed data. Red color highlights the 4 strongest correlations or the 4 largest effects among the 20 amino acids and, if applicable, their significance.

**Table 6:** Respiratory chain amino acid usage in aerobic versus facultatively anaerobic helminths (paradigm V).

Amino Acid	Aerobicity (Paradigm V)	
	Ratio	*p*
A	**1.77**	6 × 10^−5^
C	**0.43**	9 × 10^−10^
D	**0.84**	1 × 10^−5^
E	**0.99**	6 × 10^−1^
F	**0.82**	8 × 10^−5^
G	**0.83**	8 × 10^−6^
H	**1.35**	4 × 10^−11^
I	**1.16**	1 × 10^−3^
K	**1.39**	5 × 10^−5^
L	**1.01**	6 × 10^−1^
M	**1.51**	2 × 10^−6^
N	**1.16**	1 × 10^−2^
P	**1.63**	5 × 10^−9^
Q	**1.77**	9 × 10^−12^
R	**1.04**	2 × 10^−1^
S	**0.93**	5 × 10^−5^
T	**1.68**	1 × 10^−9^
V	**0.58**	3 × 10^−12^
W	**1.03**	6 × 10^−1^
Y	**0.68**	2 × 10^−11^

*p* Values derived from ANOVA on ranks analyses. Red color highlights the 4 most pronounced effect sizes among the 20 amino acids and their significance.

**Table 7:** Amino acid usage in aerobic versus obligately anaerobic bacteria and archaea.

Amino Acid	Archaea (Paradigm VI)		Gram-Positive Bacteria (Paradigm VII)		Gram-Negative Bacteria (Paradigm VIII)	
	Ratio	*p*	Ratio	*p*	Ratio	*p*
A	**1.31**	5 × 10^−2^	**1.06**	6 × 10^−1^	**1.11**	2 × 10^−1^
C	**0.73**	2 × 10^−2^	**0.53**	1 × 10−6	**0.94**	7 × 10^−1^
D	**1.23**	4 × 10^−1^	**0.96**	2 × 10^−1^	**1.01**	9 × 10^−1^
E	**0.94**	2 × 10^−1^	**0.99**	1 × 10^+0^	**0.87**	5 × 10^−3^
F	**0.91**	2 × 10^−1^	**0.99**	9 × 10^−1^	**0.95**	1 × 10^−1^
G	**1.10**	2 × 10^−2^	**1.04**	6 × 10^−1^	**0.99**	7 × 10^−1^
H	**1.07**	3 × 10^−1^	**1.06**	1 × 10^−1^	**1.11**	8 × 10^−3^
I	**0.77**	2 × 10^−2^	**0.92**	6 × 10^−1^	**0.90**	1 × 10^−1^
K	**0.60**	3 × 10^−3^	**0.89**	5 × 10^−1^	**0.85**	3 × 10^−1^
L	**0.97**	3 × 10^−1^	**1.06**	4 × 10^−3^	**1.03**	1 × 10^−1^
M	**0.90**	7 × 10^−2^	**0.87**	2 × 10^−2^	**0.96**	4 × 10^−1^
N	**0.77**	3 × 10^−2^	**0.94**	6 × 10^−1^	**1.02**	8 × 10^−1^
P	**1.09**	8 × 10^−2^	**1.10**	6 × 10^−1^	**1.04**	6 × 10^−1^
Q	**1.07**	2 × 10^−1^	**1.05**	5 × 10^−1^	**1.33**	8 × 10^−5^
R	**1.24**	3 × 10^−2^	**1.06**	7 × 10^−1^	**0.99**	8 × 10^−1^
S	**1.03**	8 × 10^−1^	**0.94**	1 × 10^−1^	**1.00**	1 × 10^+0^
T	**1.16**	5 × 10^−2^	**1.07**	1 × 10^−2^	**1.02**	4 × 10^−1^
V	**1.12**	2 × 10^−2^	**1.04**	3 × 10^−1^	**1.01**	8 × 10^−1^
W	**1.06**	6 × 10^−1^	**1.11**	3 × 10^−1^	**1.18**	2 × 10^−3^
Y	**0.92**	5 × 10^−1^	**0.91**	4 × 10^−1^	**0.91**	4 × 10^−1^

*p* Values derived from ANOVA on ranks analyses. Red color highlights the 4 largest effect sizes among the 20 amino acids and, if applicable, their significance.

**Table 8:** Ranking of amino acid usage alterations in five paradigms of oxidative stress in animals.

Amino Acid	Peroxisome (I)	Mitochondrion (II)	Respiratory Chain (III)	Longevity (IV)	Aerobicity (V)
A	14	5	7	5	2 *
C ^#^	2 *	1 *	1 *	1 *	1 *
D	10	13	12	20	13
E	9	11	8	16	19
F	7	12	15	3 *	11
G	13	7	10	7	12
H	17	9	11	8	10
I	1 *	6	19	14	15
K	19	18	17	12	9
L	16	16	20	17	20
M ^#^	8	3 *	2 *	10	7
N	18	8	4 *	15	14
P ^#^	3 *	4 *	16	6	6
Q ^#^	6	14	18	4 *	3 *
R	15	20	3 *	18	17
S ^#^	4 *	2 *	14	13	16
T	20	19	9	2 *	5
V	5	10	5	9	4 *
W	12	17	6	11	18
Y	11	15	13	19	8

Ranks were determined by sorting the averaged amino acid usage ratios of all 20 animal species (86 species in paradigm V) by decreasing distance to unity (factor 1, indicating unchanged amino acid usage), as individually determined in Table 2, Table 4 and Table 6. Distances higher than 1 or lower than 1 were treated equally, but the former were labeled in green (denoting increased amino acid usage), whereas the latter were labeled in red (denoting decreased amino acid usage). Aspects of statistical significance were not considered in this merely relative ranking. Asterisks indicate that a specific amino acid was among the four most altered amino acids within a paradigm. Hash signs in the left column highlight amino acids that belonged to the latter asterisk-labeled group in at least two paradigms, namely the amino acids C, M, P, Q, and S.

**Table 9:** Ranking of amino acid usage alterations in bacteria and archaea.

Amino Acid	Archaea (VI)	Gram-Positive Bacteria (VII)	Gram-Negative Bacteria (VIII)
A	3 *	11	7
C ^#^	2 *	1 *	9
D	7	17	16
E	17	20	4 *
F	12	19	10
G	11	18	18
H	15	9	5
I	4 *	7	6
K ^#^	1 *	3 *	3 *
L	19	14	13
M	10	2 *	12
N	5	13	14
P	13	5	11
Q	16	15	1 *
R	6	10	17
S	20	12	20
T	8	8	15
V	9	16	19
W ^#^	18	4 *	2 *
Y	14	6	8

Ranks were determined by sorting the averaged usage ratios of all sampled prokaryotes by decreasing distance to unity (factor 1, indicating unchanged amino acid usage), as individually determined in Table 7. Asterisks indicate that a specific amino acid was among the four most altered amino acids within a paradigm. Hash signs in the left column highlight amino acids that belonged to the latter asterisk-labeled group in at least two paradigms, namely the amino acids C, K, and W. Further details are provided in the legend of Table 8.

**Table 10:** Frequency changes of the four primary redox-active amino acids in all investigated paradigms (in %).

Amino Acid	Perox (I)	Mito (II)	RC (III)	Long (IV)	Aero (V)	Arch (VI)	GPB (VII)	GNB (VIII)
C	−14	−26	−59	−47	−57	−27	−47	−6
M	+11	+16	+114	−12	+51	−10	−13	−4
W	+10	+5	+81	−9	+3	+6	+11	+18
Y	+10	+6	+33	−2	−32	−8	−9	−9

Abbreviations denote: Perox, peroxisome; Mito, mitochondrion; RC, respiratory chain; Long, longevity; Aero, aerobicity; Arch, archaea; GPB, Gram-positive bacteria; GNB, Gram-negative bacteria.

## Data Availability

Data is contained within the article. Customized Perl scripts are available from the authors upon request.
